# Evolutionarily consistent families in SCOP: sequence, structure and function

**DOI:** 10.1186/1472-6807-12-27

**Published:** 2012-10-18

**Authors:** Ralph B Pethica, Michael Levitt, Julian Gough

**Affiliations:** 1 Department of Computer Science, University of Bristol, The Merchant Venturers Building, Room 3.16, Woodland Road, Bristol, UK; 2 Department of Structural Biology, Stanford University School of Medicine, Stanford, 94305, CA, USA

## Abstract

**Background:**

SCOP is a hierarchical domain classification system for proteins of known structure. The superfamily level has a clear definition: Protein domains belong to the same superfamily if there is structural, functional and sequence evidence for a common evolutionary ancestor. Superfamilies are sub-classified into families, however, there is not such a clear basis for the family level groupings. Do SCOP families group together domains with sequence similarity, do they group domains with similar structure or by common function? It is these questions we answer, but most importantly, whether each family represents a distinct phylogenetic group within a superfamily.

**Results:**

Several phylogenetic trees were generated for each superfamily: one derived from a multiple sequence alignment, one based on structural distances, and the final two from presence/absence of GO terms or EC numbers assigned to domains. The topologies of the resulting trees and confidence values were compared to the SCOP family classification.

**Conclusions:**

We show that SCOP family groupings are evolutionarily consistent to a very high degree with respect to classical sequence phylogenetics. The trees built from (automatically generated) structural distances correlate well, but are not always consistent with SCOP (hand annotated) groupings. Trees derived from functional data are less consistent with the family level than those from structure or sequence, though the majority still agree. Much of GO and EC annotation applies directly to one family or subset of the family; relatively few terms apply at the superfamily level. Maximum sequence diversity within a family is on average 22% but close to zero for superfamilies.

## Background

Proteins are made up of domains. Protein domains in this context can be regarded as the building blocks of proteins, and the smallest units of protein evolution. A small protein may consist of a single domain, larger proteins maybe contain multiple domains. A domain can be defined as a protein unit which is seen in nature either on its own or in combination with other different domains.

Detecting the evolutionary relationship between two or more domains using sequence information alone is often not possible, as sequences often diverge beyond the point of detection by comparison methods. Lack of sequence information does not necessarily show that there is no relationship between domains. If the three dimensional structure of the domains is known, evolutionary relationships can usually be recognised. The Structural Classification of Proteins (SCOP) [1-3], is a hierarchical classification system of proteins for which atomic resolution three dimensional structures are known; units in SCOP are protein domains. The SCOP classification takes protein structures published in the Protein Data Bank (PDB) [4] as the primary data source from which the domain classification is derived. The classification of domains is based on both manual curation and automatic methods, the balance of which has resulted in a classification system which is regarded as the ‘gold standard’, and is an essential bioinformatics resource.

Levels of classification in SCOP from the top down are: class, fold, superfamily, family. A class is just a convenient grouping, e.g. domains containing only alpha-helices. Folds and superfamilies have a clear and precise definition of what they are supposed to represent: a fold groups together domains which have the same topological arrangement of secondary structure; a superfamily groups together domains which share a common evolutionary ancestor. The family level sub-groups domains within a superfamily, but unlike the other levels lacks a precise definition. The first SCOP paper [1] states 30% sequence identity between members of a superfamily as significant support for a family grouping. However, in the first release of SCOP there were far fewer protein structures available (a total of 13073 domains), and selecting an arbitrary sequence identity cutoff was possible. There are now nearly ten times the number of domains (110800 as of SCOP 1.75). The family level of the classification further draws on structure and functional information in the absence of strong sequence similarity, but the meaning and the properties of the family object in SCOP remains unclear.

Many projects have been based on the SCOP classification leading to several thousand citations [5-8]. Most of these projects make use of the clear evolutionary definition of a domain, and of a superfamily, so a better understanding of the family level will add value to future work which makes use of SCOP, and enable new research questions to be addressed. The research presented in this paper was carried out in order to elucidate the meaning and significance of the SCOP family level, in particular with regard to sequence, structure and function and their relationships to family classification.

We also draw on protein functional information taken from gene ontology (GO) terms [9]. GO is a standardised vocabulary for depicting gene products in three biological concepts: Biological Process, Molecular Function and Cellular Component. Since many proteins are enzymes Enzyme Commission (EC) [10] numbers can also aid in the understanding of protein function.

## Results and discussion

To understand the meaning of a family, we compared the groupings of domains in SCOP to determine the similarity to automatically generated groupings based independently on the three aspects we wished to investigate: sequence, structure and function. Since we begin without a pre-conceived idea of the granularity or size/depth of the groupings it is necessary to generate the automatic groupings at every possible level. This is represented by a tree which is the result of hierarchical clustering of the domains based on one of the three sources of information: sequence similarity, structural similarity, functional labels (in the forms of Gene Ontology and Enzyme Classification). The level of agreement between one type of information and the grouping of a SCOP family can be assessed by asking whether each edge in the tree divides domains into family groups, or splits a family, grouping together domains from different families.

**Figure 1:**
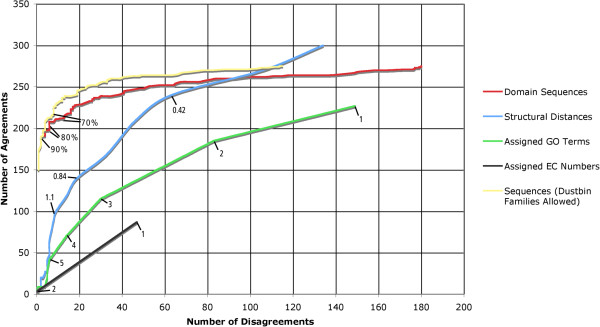
**The number of superfamily agreements/disagreements with SCOP for varying confidence values.** A ROC curve showing the number of superfamilies containing agreements against the number containing disagreements of trees with SCOP's groupings, for confidence values decreasing from left to right. For sequence trees, confidence is based on the bootstrap value assigned to an edge. Structures are ranked using the total structural distance, and function is ranked by the total number of GO terms or EC numbers which support an edge.

The ROC curve Figure 1 shows the number of disagreements/agreements of the trees produced from sequence, structure and functional data with the SCOP family classification for varying confidence values. For sequence, confidence is ranked by bootstrap percentages, for structural data the confidence is based on the structural distance scores, and for function, confidence is based on the total number of terms which suggest a particular clade in the trees. See materials and methods for details of a web resource providing all data and trees.

### Sequence

Within the literature there is variation in suggested levels for the minimum informative bootstrap confidence [11,12], with most suggesting about 70-80% required for confidence. We found that from 2046 families across 428 superfamilies, 99.6% of the phylogenetic trees agree with the SCOP groupings for bootstrap values above 80%. We also found that, although less reliable, there is useful information which can be acquired from the trees for bootstrap values down to 60%. These results show that, to the extent to which sequence information can reliably determine evolutionary relationships, SCOP family groupings are evolutionarily consistent. Classical sequence phylogenetics are quite reliable for high bootstrap values, but are limited in the evolutionary distance over which they can resolve relationships. There are plenty of SCOP family groupings which sequence-based phylogenetics alone is unable to determine with high confidence - the low confidence parts of the tree. Although the classical phylogenetic analysis cannot inform us directly about the evolutionary consistency of many family groupings, the fact that there is such strong agreement with those that it can, gives a strong suggestion that the others (classified independently from this information) are also likely to be evolutionarily consistent.

**Figure 2:**
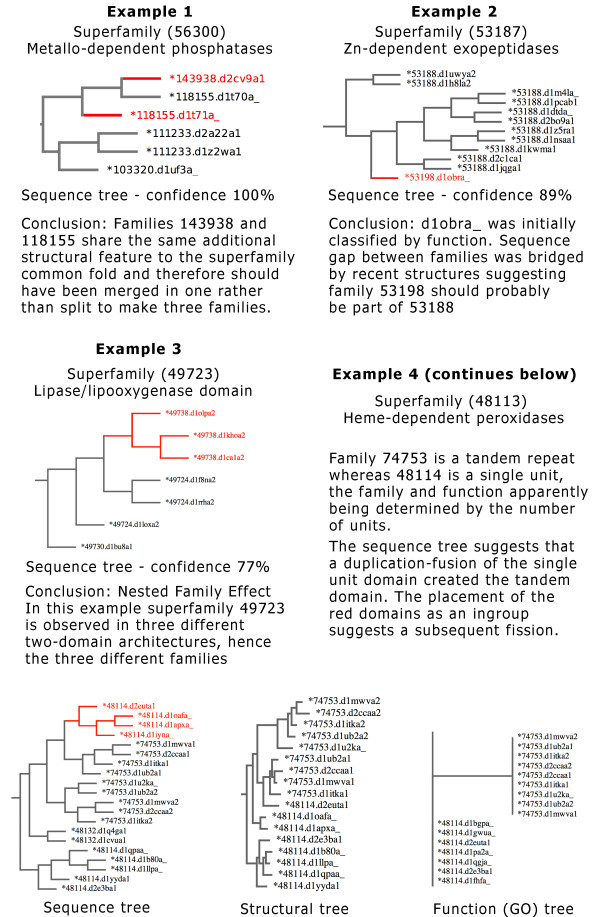
**Examples of disagreements with SCOP.** Examples of SCOP superfamilies which contain a disagreement found with trees based on sequence information, supported by high confidence values. Four of the common reasons for disagreement are explained. Images produced with TreeVector [14].

The top 13 edges which conflicted with the sequence trees were examined. These are shown in a table in Figure 2, along with an example of each type of disagreement. The most frequent disagreement was from families which were classified not long after the creation of SCOP. These families were classified at a time when PFAM [13] sequence data was not available, and therefore did not provide evidence in the curation of SCOP families. Sequence information from PFAM is now a contributing factor of data used to guide the classification. An example is shown in Example 1. We also find examples such as that shown in Example 2, where a family has been decided in SCOP based on function. Trees based on both sequence and structure place the single domain Pancreatic carboxypeptidases family between domains for a different family causing a disagreement of the trees with SCOP families. In this case the classification of a domain into a new family of its own was likely based on a functional signal, however the tree based on function places the domain in a similar way to that of structure and sequence suggesting the domain should probably belong to the surrounding family. Our method classes 'nested families' as inconsistent with evolution (shown in Example 3), whereby one family grows from another in the tree. In some sense this is more a reflection of the limited number of levels in the hierarchy, suggesting that there are some families that actually represent a 'sub' family of another. We also find a small number of other artefacts, where is a family classification based on the source species. This is can happen with proteins found in viruses. We also see cases such as duplications of domains grouped within the same family, an illustration of this is shown in Example 4.

A potential factor which contributes to the disagreements seen in trees calculated from sequence data compared to those from the other data sources is also worth noting. Diverse superfamilies with very low sequence identity between member domains may provide an unreliable multiple sequence alignment thereby creating a result tree with limited accuracy. Anomalies introduced from this effect are more likely to be seen in very large superfamilies with a great deal of structural variation.

### Structure

The trees built from automatically generated structural distances largely agree, but are not always consistent with SCOP’s hand annotated groupings. The hand classification of structures in SCOP at the superfamily and fold levels is often referred to as the gold standard in the field, and clearly surpasses any fully automatic method. Since detectable structural similarity remains long after sequences have diverged beyond the point of recognition, the structurally-derived trees are able to resolve deeper edges of the tree with higher confidence than the sequence-based ones (the intersection of the red and blue lines in Figure 1). That the trees are largely in agreement with the family classification indicates that SCOP is also evolutionarily consistent at greater divergence distances. The differences we see could either be cases where SCOP has grouped domains based on some criterion other than evolution (e.g. common function), or may be due to geometric structural distance being in some cases a poor measure of divergence. For some proteins, changes to the structure of a binding site may be the best indication of evolutionary divergence, but these changes make a relatively small contribution to the automatic superposition of the whole body. Conversely, movements of secondary structures relative to each other, e.g. a change of angle between beta-sheets [15], can cause dramatic changes in superposable structural distance which mask the true relationships. In this way structural geometric distance does not always equate to evolutionary distance.

Examining high ranking disagreements between the SCOP family classification and structural trees can mostly be explained by the above, however one exception is shown in Example 2 from Figure 2. This example shows a sequence tree but we see the same disagreement when we look at the structural tree, and so in this case it suggests the possibility of a mis-classification.

### Function

The lines for EC numbers and GO terms shown in Figure 1 are smaller and less smooth than the others. This is because confidence values are generated using the total number of independent features that support a particular edge of the tree. There are not very many GO features per tree and barely any for EC number. This is partly due to a lack of richness in the ontological hierarchy but also due to the incompleteness of the annotation of the domains with terms. Trees derived from both GO and EC functional data are less consistent with the family level than trees derived from structure or sequence, though the majority still agree with the classification. This may be due to the low quality of the derived functional dataset, most commonly the lack of functional annotation for a particular domain. Functions are also appended to the protein chain rather than individual domains, therefore terms may be uninformative for two domains found within the same protein. The fact that the correlation with function is so much weaker than sequence and structure suggests that although function may guide the choice of granularity or level of grouping of families in SCOP (see section on Distribution of GO terms), it is not a primary source of information for determining relationships.

### Dustbin families

In SCOP all domains must belong to a family, so a superfamily with a single member must also have a single family. As more structures are added to a superfamily over time, there may be new additions that have enough in common to group them apart from the rest and a second family is created to hold them. If this happens successively the result is that some families contain domains with something in common, but any leftovers lacking common features with each other may remain in the original family that contained the first member of the superfamily. These non-specific families are referred to here as 'dustbin families'. The 'dustbin families' line in Figure 1 is derived from the same trees as for the standard domain sequences line, but the rules by which edges are defined as conflicting are adjusted to not penalise for the presence of a single dustbin family in each superfamily. Remarkably, despite expectations, the results show that they are not a major feature of the SCOP classification.

### Sequence identity

**Figure 3:**
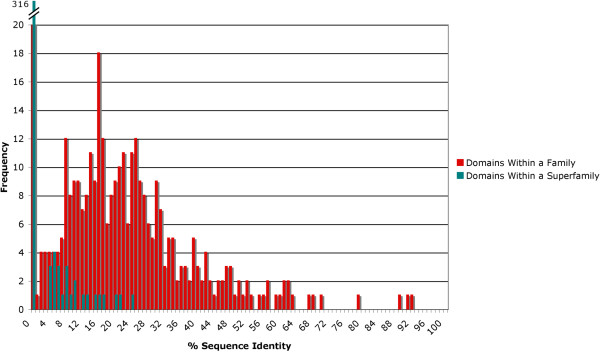
**Sequence divergence in families and superfamilies.** Graph shows the maximum sequence diversity between two members of the same superfamily (or family) in SCOP. Domains which continue to diverge beyond detectable sequence identity have their distribution collapsed to the far left side of the graph; the large number with zero percent sequence identity represent those cases in which BLAST was unable to find alignment.

Figure 3 shows the maximum sequence divergence between any two members of a family or superfamily, i.e. a measure of the divergence within the family or superfamily. The analysis of sequence distances shows that the maximum sequence diversity for domains grouped within a family is on average 22% with the majority of families having a maximum sequence distance of 10-30%. Superfamilies on the other hand have a sequence diversity spread of 8% and below, with the average being close to zero. While it is well known that remote homology detection at the superfamily level is a difficult problem, the data show that about half (169) of the 341 families (the most divergent family within each of the 341 superfamilies in the analysis) contain members with no less than 20% sequence identity.

**Figure 4:**
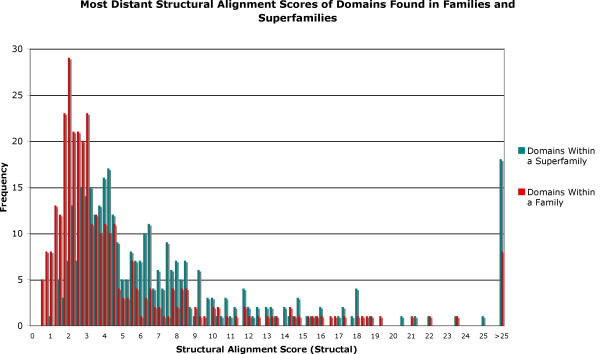
**Structural divergence in families and superfamilies.** Graph shows the maximum structural diversity between two members of the same superfamily (or family) in SCOP. Structural distances used are the scores produce by Structal for the alignment of two domains.

Figure 4 shows the maximum structural distance found between two members of the same superfamily or family. The distribution shows that the maximum structural distances are greater between two members of the same superfamily than to two domains grouped in the same family.

It is clear from the distribution in the graph in Figure 3 that SCOP families are not selected by simply choosing a random sequence identity cutoff, and that the process of curation is much more elaborate.

### Distribution of GO terms

**Figure 5:**
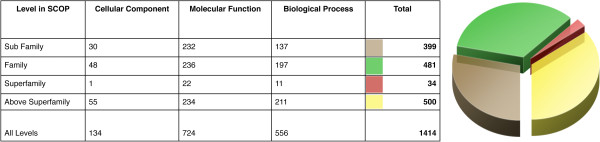
**Level in SCOP of all single domain proteins associated with a specific GO term.** Figure shows the level in SCOP at which all single domains associated with a particular GO term are found. I.e. if the group represents a family or superfamily. These are also broken down into the three main ontologies of GO terms.

Figure 5 shows the distribution of GO terms annotated to single domains across SCOP. We see that approximately 1/3 of GO and EC annotation applies directly to one family, another 1/3 to a subset of a family, and the remaining 1/3 scattered across multiple superfamilies, with strikingly few terms that apply at the superfamily level. One would expect that the terms in the sub-family would be lower down the GO hierarchy and those spanning multiple superfamilies would be broader terms found higher up the hierarchy, but the distribution across the GO hierarchy is quite similar in each of the three major segments of the pie chart shown in Figure 5. This distribution does not change significantly when looking at each of the three ontologies of GO (molecular function, cellular localisation, biological process) separately. A more detailed view is shown in Additional file 1: Table S1 in additional files.

Despite the weak link between SCOP family classification and the edges of trees representing functional data, we see a very large proportion of functional terms corresponding to exactly one family, and almost none close to the superfamily level. This suggests that the relationships between members of a superfamily and their distance apart is evolutionary, having been based on evidence from structure and sequence (not function), but the granularity at which to divide the members of a superfamily is decided by function. I.e. domains are not grouped based on their function, but the number of groups relates to the number of functions.

## Conclusions

Sequence information contributes to the classification of domains into families, but alone is not enough. To classify a family evolutionarily: it must be consistent with sequence phylogenetics, will likely draw on structural distance, and will often coincide with a particular function. Sequence diversity between families (within a superfamily) is considerably greater than within a family. Sequence phylogenetics do not give a strong enough signal at the superfamily level to classify families, but where there is a signal it is consistent with the SCOP classification. Structural information is necessary for identifying evolutionary relationships of families in a superfamily where sequence identity is low. We see that although function does not determine the relationships, i.e. edges, it is used to guide the level at which the tree is cut to make a family, i.e. the choice of node from which to derive a clade (granularity).

The families in SCOP represent a level at which sequence, structure, function plus other information on a shared peculiarity must all be taken into account. A balance of the strengths of signals available is used to establish the evolutionary relationships and resolve the groupings.

## Methods

The data for all trees used to generate Figure 2 are available as a web resource at http://supfam2.cs.bris.ac.uk/pethica/scopresults. The data may be ranked on each of the confidence scores separately or together. For every superfamily there are tree images for sequence, structure and function annotated with the PDB domain and SCOP family ID as shown in Figure 2. The tree data can additionally be downloaded in Newick format [16]. Also available are all the matrices of Structal [17] data used to generate the structural distance trees.

### Sequence based phylogeny

Domain sequences for SCOP version 1.73, filtered to 95% sequence identity were obtained from ASTRAL [18]. The complete set of sequences was filtered to remove superfamilies for which SCOP's family level classification could not be contested. These cases included superfamilies containing a single family, those where each family contained only one member, and any superfamily made up of three or less domains. A detailed breakdown of the number of domains, families, and superfamilies used in the analysis can be found in Additional file 2: Table S2.

For each superfamily in the classification the sequences of assigned domains were used to produce an alignment using MUSCLE [19]. Alignments were converted to Stockholm format using sreformat which is part of the HMMER package [20]. Quicktree [21], a fast implementation of the neighbour joining algorithm was used to produce runs of both 300 and 600 bootstrap replicate trees from the sequence alignments. Phylip Consense was used to create a single consensus tree from the sets of replicate trees. In this process, the number of occurrences of a particular edge from the replicate trees was converted to a single confidence score giving the final tree confidence values for each edge.

A second set was also produced where domain sequences were padded with homologue sequences from the SUPERFAMILY database. These were aligned, and trees created as with the original set. A script was used to remove the homologues from the trees leaving only the original domain sequences, but preserving all phylogenetic relationships. The dataset calculated without homologues, with 300 replicates was chosen as very little difference was seen between the two replicate sets, and the addition of homologues sequences created larger alignments which were handled badly by the phylogenetic algorithms.

### Structural phylogeny

PDB style protein three dimensional structures for the same filtered SCOP 1.73 set of domains were taken from ASTRAL. The same filtered set of SCOP 1.73 domains as for sequence was used. Structal [17] was used to compare the 3D structures of every domain against every other in a superfamily, for all superfamilies in the set in a computationally expensive process of around 1.5 million structural comparisons. The Structal software was chosen from the large number of other structural comparison methods due to its balance of speed and accuracy for a computation of this kind. The Structal SAS scores (100*RMS/Number of positions matched) for each domain were used to create a matrix of structural distances for each superfamily. The neighbour joining algorithm in the PAUP [22] package was used to compute phylogenetic trees from the distance matrices.

### Functional phylogeny

Gene ontology (GO) data from EBI GOA [23] was used to annotate domains with functional terms using the same set of domains that was used for the sequence and structure trees. For each superfamily, a binary presence/absence matrix was generated of all GO terms versus all domains in the superfamily. The terms were treated independently of the hierarchy, but uninformative terms (present in all or present in only one domain) were ignored. For each superfamily the presence/absence matrix was used to generate a phylogenetic tree using PAUP neighbour joining. An additional set of functional trees was also generated using the same technique, but with functional data from Enzyme Commission (EC) numbers.

### Testing against SCOP families

Phylogenetic trees of domains in each superfamily produced by each method could then be compared with the groupings at the SCOP family level. An algorithm was produced to traverse the trees and identify if a particular edge agreed, disagreed or was uninformative with regard to SCOP families:

An edge of the tree is said to agree with SCOP if one side contains the full set of domains for a certain family and no members of another family.

An edge disagrees with SCOP when domains from a certain family are found on both sides along with domains from a different family.

A neutral or uninformative edge is where one side contains only members from a certain family, but not the complete set. i.e. more are found on the other side of the edge.

**Figure 6:**
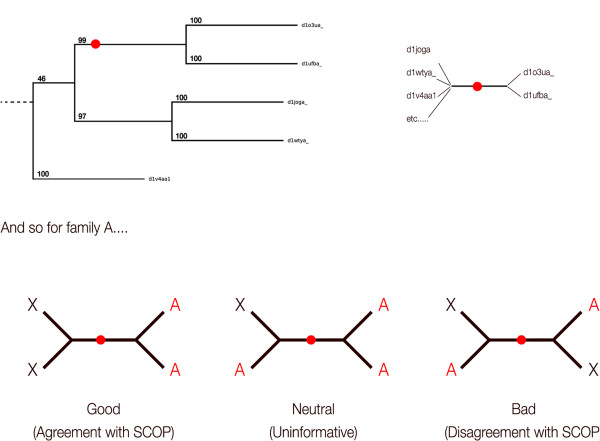
**An overview of the algorithm used to determine agreements/disagreements of trees with SCOP's groupings.** Figure shows part of a tree built from domain sequences in a SCOP superfamily, and illustrates the algorithm involved in establishing if the tree agrees or disagrees with SCOP's family level grouping.

An overview of the algorithm used is shown in Figure 6.

### Sequence divergence of domains in superfamilies and families

Sequences for domains in SCOP 1.73 superfamilies were acquired from ASTRAL. Superfamilies containing a single domain only were removed. For each superfamily grouping, sequence identities were sequentially calculated with Washington University BLAST [24], the highest sequence identity members being removed until only the two most distant sequences remained. This process was repeated for domains grouped in families to give sequence distance scores for all relevant families and superfamilies in SCOP.

### Functional divergence across SCOP

For each GO term in the EBI GOA dataset a list of single domain proteins with the particular annotation was generated. The sequence identity of the two most distant sequences in the set was determined. The distribution of domains across the SCOP classification and level in the hierarchy for a specific functional annotation was also calculated. e.g. All domains contained within a specific family or superfamily.

## Competing interests

The author(s) declare that they have no competing interests.

## Authors' contributions

Both JG and RP designed the study, ML advised at a later stage of the study. RP carried out most of the work, ML carried out modifications to STRUCTAL. RP and JG were involved in the preparation of the manuscript. All authors read and approved the final manuscript. ML is supported by NIH GM063817 and is the RW and VKC Professor of Cancer Research. JG is supported by BBSRC grant (BB/G022771/1).

## Supplementary Material

Additional file 1: Table S1. An Extended View of the Levels at which All Single Domain Proteins Associated With a Specific GO Term are Found in SCOP. Table shows an extended version of the level in SCOP at which all single domains associated with a particular GO term are found. The different levels shown are designed to illustrate the level at which GO terms fall, independently to the SCOP hierarchy. The distribution of GO terms assigned to domains is broken down into the following categories: *· Sub Family*: GO terms are found in some, but not all members of a family. *· Family Equivalent*: Exactly fits a family, i.e. found in all members of a family, but not in other families. *· Multi Family*: Found in all members of more than one family, but not in all families in the superfamily. *· Partial Family*: Completes one or more families, is absent from one or more families and is incomplete from exactly one family of a specific superfamily. *· Scattered Families*: May or may not complete one family, more than one incomplete family, and at least one empty family per superfamily. *· Scattered in Superfamily*: Present in, but does not complete, all families in a superfamily. *· Almost Superfamily*: Present in all families, competes some of them. *· Superfamily Equivalent*: Present for every domain of just one superfamily. *· Multi Superfamilies*: Present in every domain in more than one superfamily. *· Partial Superfamilies*: Completes at least one superfamily; partially completes exactly one other superfamily. *· Scattered Superfamilies*: May or may not complete one superfamily; present but not completing at least one other superfamily. Click here for file

Additional file 2: Table S2. Statistics for the number of domains used in the phylogenetic analysis. Statistics for domains in raw SCOP 1.73 and after filtering to 95% sequence identity and removal of trivially solvable cases. Click here for file
