# Supplementary material for: Evolutionarily consistent families in SCOP: sequence, structure and function
Source: BMC Struct Biol. 2012 Oct 18;12:27. doi: 10.1186/1472-6807-12-27 (PMC3495643; doi:10.1186/1472-6807-12-27)
Supplement: Additional file 1: Table S1. — An Extended View of the Levels at which All Single Domain Proteins Associated With a Specific GO Term are Found in SCOP. Table shows an extended version of the level in SCOP at which all single domains associated with a particular GO term are found. The different levels shown are designed to illustrate the level at which GO terms fall, independently to the SCOP hierarchy. The distribution of GO terms assigned to domains is broken down into the following categories: · Sub Family: GO terms are found in some, but not all members of a family. · Family Equivalent: Exactly fits a family, i.e. found in all members of a family, but not in other families. · Multi Family: Found in all members of more than one family, but not in all families in the superfamily. · Partial Family: Completes one or more families, is absent from one or more families and is incomplete from exactly one family of a specific superfamily. · Scattered Families: May or may not complete one family, more than one incomplete family, and at least one empty family per superfamily. · Scattered in Superfamily: Present in, but does not complete, all families in a superfamily. · Almost Superfamily: Present in all families, competes some of them. · Superfamily Equivalent: Present for every domain of just one superfamily. · Multi Superfamilies: Present in every domain in more than one superfamily. · Partial Superfamilies: Completes at least one superfamily; partially completes exactly one other superfamily. · Scattered Superfamilies: May or may not complete one superfamily; present but not completing at least one other superfamily. [file 1472-6807-12-27-S1.pdf]

Cellular Component	Molecular Function	Biological Process	Total	Title	Description<br>A particular GO term...
30	232	137	399	Sub Family	Appears in 1 family in 1 superfamily, but not all members
48	231	193	472	Family Equivalent	Is in all members of just one family in 1 superfamily
0	5	4	9	Multi Family	Is in all members of more than one family, but not all families in a superfamily
0	8	4	12	Partial Family	Completes 1 or more families and has exactly 1 incomplete family and 1 or more empty in 1 superfamily
0	10	1	11	Scattered Families	May or may not complete 1 family, more than one incomplete family and at least 1 empty family in 1 superfamily
0	0	0	0	Scattered in Superfamily	Is present but does not complete all families in 1 superfamily
0	2	1	3	Almost a Superfamily	Is present in all families, completes some of them
1	2	5	8	Superfamily Equivalent	Present in every domain in just one superfamily
7	17	10	34	Multi Superfamily	Present in every domain in more than one superfamily
13	44	45	102	Partial Superfamilies	Completes at least one superfamily, Partially completes exactly one other superfamily
35	173	156	364	Scattered Superfamilies	May or may not complete 1 superfamily, present, but not completing more than one other superfamily
**134**	**724**	**556**	**1414**		
