# Supplementary material for: Evolutionarily consistent families in SCOP: sequence, structure and function
Source: BMC Struct Biol. 2012 Oct 18;12:27. doi: 10.1186/1472-6807-12-27 (PMC3495643; doi:10.1186/1472-6807-12-27)
Supplement: Additional file 2: Table S2. — Statistics for the number of domains used in the phylogenetic analysis. Statistics for domains in raw SCOP 1.73 and after filtering to 95% sequence identity and removal of trivially solvable cases. [file 1472-6807-12-27-S2.pdf]

Data	Superfamilies	Families	Domains
Unfiltered SCOP 1.73	1,777	4,464	97,178
95% Filtered & trivial cases removed	341	1,875	10,346
